# Omics profiles of fecal and oral microbiota change in irritable bowel syndrome patients with diarrhea and symptom exacerbation

**DOI:** 10.1007/s00535-022-01888-2

**Published:** 2022-07-30

**Authors:** Yukari Tanaka, Riu Yamashita, Junko Kawashima, Hiroshi Mori, Ken Kurokawa, Shinji Fukuda, Yasuhiro Gotoh, Keiji Nakamura, Tetsuya Hayashi, Yoshiyuki Kasahara, Yukuto Sato, Shin Fukudo

**Affiliations:** 1 Department of Gastroenterology, Sendai Kousei Hospital, Sendai, Japan; 2 Department of Behavioral Medicine, Tohoku University Graduate School of Medicine, 2-1 Seiryo, Aoba, Sendai 980-8575 Japan; 3 Division of Translational Informatics, Exploratory Oncology Research and Clinical Trial Center, National Cancer Center, Chiba, Japan; 4 Tohoku Medical Megabank Organization, Tohoku University, Sendai, Japan; 5 Advanced Genomics Center, National Institute of Genetics, Shizuoka, Japan; 6 Institute for Advanced Biosciences, Keio University, Yamagata, Japan; 7 Gut Environmental Design Group, Kanagawa Institute of Industrial Science and Technology, Kanagawa, Japan; 8 Transborder Medical Research Center, University of Tsukuba, Ibaraki, Japan; 9 Department of Bacteriology, Faculty of Medical Sciences, Kyushu University, Fukuoka, Japan; 10 Department of Fetal and Maternal Therapeutics, Tohoku University Graduate School of Medicine, Sendai, Japan; 11 Research Laboratory Center, Faculty of Medicine, University of the Ryukyus, Nishihara, Okinawa Japan

**Keywords:** Microbiome, 16S rRNA gene, Metatranscriptome, Metabolome

## Abstract

**Background:**

Irritable bowel syndrome (IBS) is a disorder of gut–brain interaction, including dysregulation of the hypothalamic–pituitary–adrenal axis with salivary cortisol changes. However, the role of gastrointestinal microbiota during IBS symptom exacerbation remains unclear. We tested the hypothesis that the microbial species, gene transcripts, and chemical composition of fecal and oral samples are altered during the exacerbation of IBS symptoms.

**Methods:**

Fecal, salivary, and dental plaque samples were collected at baseline from 43 men with IBS with diarrhea (IBS-D) and 40 healthy control (HC) men. Samples in the IBS-D patients were also collected during symptom exacerbation. The composition of the fecal microbiota was determined by analyzing the 16S rRNA gene, RNA-based metatranscriptome, and metabolites in samples from HC and IBS patients with and without symptom exacerbation. Oral samples were also analyzed using omics approaches.

**Results:**

The fecal microbiota during IBS symptom exacerbation exhibited significant differences in the phylogenic pattern and short-chain fatty acid compared with fecal samples during defecation when symptoms were not exacerbated. Although there were no significant differences in the phylogenic pattern of fecal microbiota abundance between HCs and IBS-D patients, significant differences were detected in the expression patterns of bacterial transcriptomes related to butyrate production and neuroendocrine hormones, including tryptophan-serotonin-melatonin synthesis and glutamine/GABA. The composition of plaque microbiota was different between HC and IBS-D patients during normal defecation.

**Conclusions:**

Our findings suggest that colonic host-microbial interactions are altered in IBS-D patients during exacerbation of symptoms. There were no overlaps between feces and oral microbiomes.

**Supplementary Information:**

The online version contains supplementary material available at 10.1007/s00535-022-01888-2.

## Introduction

Irritable bowel syndrome (IBS) is a chronic gastrointestinal disorder characterized by recurrent abdominal pain and changes in defecation frequency and/or the form of stools [1]. Aberrant gut microbiome and/or host responses may influence IBS pathophysiology [2]. However, the role of gut microbiota in IBS development remains circumstantial and controversial [3]. IBS pathophysiology is likely a disorder of the brain-gut axis [4]. Neurotransmitters produced by colonic microbiota influence gut motility and sensitivity [5, 6]. Increased production of microbial metabolites promotes peripheral serotonin release from colonic enterochromaffin cells (ECs) [5]. The microbes accelerate enteric glutamate-GABA circuits [6], resulting in receptor stimulation, including 5-HT3 receptors [7]. Antagonizing 5-HT3 receptors improves symptoms of IBS with diarrhea (IBS-D) [8]. Moreover, short-chain fatty acids (SCFAs) produced by colonic bacterial fermentation influence colonic motility and sensitivity via the sympathetic nervous system [9]. Although the metabolomic response of individual gut microbes has been investigated, the community-wide host-microbiome relationship, especially during IBS symptom exacerbation, is poorly understood.

We reported that the serum cortisol response to exogenous administration of corticotropin-releasing hormone (CRH) is altered in patients with IBS during intense colorectal distention [10]. Hypothalamic CRH secretion induces adrenocorticotropic hormone (ACTH) secretion from the pituitary gland, which stimulates cortisol release from the adrenal gland [11]. Patients with IBS exhibit enhanced serum as well as salivary cortisol responses to gastrointestinal stimulation [11, 12]. The enhanced cortisol response may enhance components of the oral microbiome.

Oral microbiome changes may be associated with chronic inflammatory diseases, including inflammatory bowel disease [13, 14]. Moreover, an overlap in the abundance and function of species between the oral and gut microbiomes was observed in colorectal cancer [15]. A decreased richness of the phylum Bacteroidetes and the genus *Bacillus* was found in the buccal mucosal microbiome of overweight IBS patients [16]. These findings suggest that the oral microbiome correlates with colonic pathophysiology.

Herein, we evaluated fecal and oral microbiota and overlaps in these microbiomes in patients with IBS. Metatranscriptomics is used to evaluate the expression and function of specific organisms over time [17]. Therefore, we conducted a metatranscriptomics analysis in addition to studying the 16S rRNA gene-based metagenome and the metabolome of fecal samples obtained from patients with IBS and healthy subjects. To investigate the response to symptom exacerbation, samples were collected from IBS subjects with and without symptom exacerbation. A number of factors, including diet, history of antibiotic intake, obesity, hyperlipidemia [18], age, and stool consistency [19], are known to affect fecal microbiome diversity. Therefore, we investigated patients with IBS-D and healthy subjects while minimizing these confounding factors. The first aim of the study was to determine changes in the fecal microbial-host relationship during symptom exacerbation in IBS-D patients. The second aim was to determine if oral microbial compositions also change during symptom exacerbation in conjunction with fecal components.

## Materials and methods

### Participants

This study included 43 male patients with IBS-D [age: mean ± standard deviation (SD), 21.8 ± 1.7 years] and 40 healthy male controls (age: 22.1 ± 1.3 years). Blood samples were collected and anthropometric measurements were made on all study participants. Subjects with BMI > 25 or < 18.5 and those with hyperlipidemia were excluded from the study. All patients with IBS-D were diagnosed according to the Rome lll criteria [20]. At the time of study entry, a gastroenterologist skilled in the treatment of IBS interviewed the participants about their symptoms. Healthy controls (HC) were subjects without symptoms of functional bowel disorders. None of the IBS-D patients or HC had organic diseases or mental disorders. The use of the following drugs was prohibited at least for the indicated week(s) before the baseline sampling; laxatives, antidiarrheals, or probiotics for one week and antibiotics, anti-inflammatory drugs, corticosteroids, proton pump inhibitors, transit modulators or tranquilizers for 6 weeks. Written informed consent was obtained from all participants before their participation. This study was approved by the Ethics Committee of the Tohoku University Hospital, Japan.

The IBS Severity Index (IBS-SI) [21], the State-Trait Anxiety Inventory (STAI) [22], and the Self-Rating Depression Scale (SDS) [23] were administered to all participants the day before the experiment to assess their anxiety and depression levels. The oral status of all subjects was determined by a dentist at the Tohoku University Hospital. Periodontal disease was defined based on pocket depths at six sites per tooth, bleeding on probing, and tooth mobility according to the World Workshop on the Classification of Periodontal and Peri-implant Diseases and Conditions [24, 25]. The patients were asked to record their numerical rating scale (NRS) of abdominal pain, abdominal discomfort, abdominal bloating, incomplete evacuation, difficulty in passing stool, or dissatisfaction on bowel movements evaluated from 1 (minimum) to 7 (maximum), Bristol Stool Form Scale from 1 (lumpy) to 7 (watery), and number of bowel movements per day for 14 days from the beginning of the study.

### Collection of fecal and oral samples

Fecal samples were collected from IBS-D patients with (IBS-s) and without (IBS-n) symptom exacerbation and HC. In accordance with Rome III diagnostic criteria, IBS-s refer to specimens on the day with abdominal pain or abdominal discomfort as well as loose or watery stools confirmed by Bristol Stool Form Scale. On the other hand, IBS-n refers to specimens on the day without the above symptoms. For the IBS patients, subsequent sampling was conducted more than 24 h apart. Oral samples were collected at the same time as the fecal samples. Oral plaques of molar teeth were sampled using a disposable toothbrush and dissolved in 5 mL of 0.9% saline, and 5 mL of saliva was collected. Samples were immediately stored at 4 °C and then frozen at − 80 °C within 12 h of collection and stored at − 80 °C until sample processing.

### DNA extraction and 16S rRNA gene sequencing

For DNA extraction from feces, dental plaques, and saliva, samples were added to tubes containing glass beads (MO BIO, Carlsbad, CA, USA) and homogenized using a Mixer Mill MM 400 (Retsch, Haan, Germany) for 10 min at 30 Hz. After a brief centrifugation at 10,000 g, the supernatant was removed. The sample homogenates (~ 0.25 mg, including the glass beads) were subjected to DNA extraction using a PowerSoil DNA Isolation Kit (MoBio/Qiagen). DNA was eluted from the spin column in 100 μL of RNase-free water and stored at − 80 °C after measuring the concentration and quality using a Nanodrop (Thermo Scientific, Wilmington, DE, USA). Next-generation sequencing library preparations and Illumina MiSeq sequencing were conducted at GENEWIZ. Amplicons were generated using 30–50 ng of DNA and a MetaVx Library Preparation kit (GENEWIZ). V3 and V4 hypervariable regions of prokaryotic 16S rRNA gene were selected for generating amplicons and for subsequent taxonomy analyses. The QIIME1 data analysis package was used for 16S rRNA data analysis. Sequences were grouped into operational taxonomic units with the clustering program VSEARCH (1.9.6) against the SILVA 128 database preclustered at 97% sequence identity. β-diversity was calculated using weighted and unweighted UniFrac and principal coordinate analyses were performed. An unweighted pair group method with an arithmetic mean (UPGMA) tree from the β-diversity distance matrix was built.

### RNA-sequencing and metatranscriptome experiments

For RNA extraction, samples were processed as described above but the phenol–chloroform-based PowerSoil RNA Isolation Kit (MoBio/Qiagen, Carlsbad, CA, USA) was used. Total RNA (3 µg) was depleted of ribosomal RNA using the Ribo-Zero Bacteria Kit (Illumina). Indexed RNA-seq libraries were prepared from the rRNA-depleted RNA using the NEBNext Ultra II Directional RNA Library Prep Kit for Illumina (New England Biolabs). Libraries were pooled and sequenced on an Illumina HiSeq 2500 platform with a single 101 bp that consequently yielded 52.6 ± 2.5 million reads/sample. Contaminating human DNA sequences were removed.

### Data analysis of metatranscriptome sequencing

Quality filtering and sequencing adapter trimming of reads were performed using fastp version 0.12.5 with parameters “-G -3 -n 1 -l 70 [26].” High-quality reads of all 45 samples (15HC, 15 IBS-n, and 15 IBS-s) were co-assembled using MEGAHIT version 0.3.3-a with parameters “–k-min 25 –k-step 10 –k-max 91 [27].” Protein-coding genes were predicted from the contig sequences using MetaGeneMark version 3.38 [28]. Transcript abundances of genes in each sample were calculated by mapping reads against the contig sequences using BWA-MEM version 0.7.12 with a default parameter [29]. Normalization of gene lengths and total read numbers were performed by calculating the transcripts per million (TPM) value for each gene in each sample [30]. Functional predictions of genes were performed by sequence identity searching against the amino acid sequence database, Kyoto Encyclopedia of Genes and Genomes (KEGG), from 2018 [31]. The identity search of protein-coding genes was conducted using MMseqs2 release 2 with parameters “-s 4 -c 0.5,” a sequence identity > 40%, and a bit score > 70 [32]. Functions for protein-coding genes were summarized using KEGG Orthology (KO) [31]. Because only a small fraction of the genes in the KEGG amino acid sequence database are KO-assigned genes, we normalized the KO abundance of a sample by dividing by the total KO-assigned gene abundance of a sample. The phylum composition of transcripts in a sample was calculated using 35 universally single-copy genes with an identity > 60% and a bit score > 70. The 56 Gut–Brain Modules (MGB) defined in Valles-Colomer et al. 2017 were used to summarize the abundance of neurotransmitter-related KOs [33].

### Fecal and saliva metabolome analyses

Because of sampling instability, fecal metabolome analysis was performed with 39 HC, 35 IBS-n, and 34 IBS-s samples and salivary metabolome analyses were performed with 38 HC, 39 IBS-n, and 40 IBS-s samples. In IBS patients, 29 pairs of stool data and 39 pairs of saliva samples were available for both with and without symptom exacerbation. Saliva samples were deproteinized with equal volumes of acetonitrile. After vortexing, the samples were centrifuged (15,000×*g*, 5 min, room temperature) and the supernatants were analyzed. UHPLC-QTOF/MS analysis was performed on an Acquit Ultra Performance LC I-class system (Waters Corp. Milford, MA, USA) connected to a Waters Synapt G2-Si QTOF MS fitted with an electrospray ionization (ESI) source operated in the negative ion mode. Samples were separated using a Waters Acquity UPLC BEH Amide Column (1.7 μm, 2.1 × 150 mm) kept at 45 °C and a flow rate of 0.4 mL/min. Acetonitrile with 10 mM ammonium bicarbonate (95:5, v/v) was used as mobile phase A, while acetonitrile with 10 mM ammonium bicarbonate (5:95, v/v) was used as mobile phase B. The gradient was applied as follows: 1% B at 0–0.1 min, 1–70% B at 0.1–6.0 min, 70–1.0% B at 6.0–6.5 min, and 1% B at 6.5–10.0 min. All data were processed using ProgenesisQI software (Nonlinear Dynamics, Newcastle, UK) for peak picking, alignment, and normalization to produce peak intensities for retention time (tR) and *m*/*z* data pairs. Features were identified from the Chemspider DB, Human Metabolome Database (HMDB), and Lipidmaps using the precursor and fragment ion spectra obtained by MS. The intensities of the identified features were imported to EZinfo software (Waters) for multivariate analysis and their relative quantities were evaluated using PCA, PLS-DA, and OPLS-DA [34].

Capillary electrophoresis time-of-flight mass spectrometry (CE-TOFMS)-based metabolome analysis of fecal samples was performed as described previously with slight modifications [35]. In brief, fecal samples were lyophilized using a VD-800R lyophilizer (TAITEC) for 24 h. Freeze-dried feces were disrupted with 3.0-mm Zirconia Beads (Biomedical Science) by vigorous shaking (1500 rpm for 10 min) using Shake Master (Biomedical Science). Fecal metabolites were extracted using the methanol:chloroform:water extraction protocol. CE-TOFMS experiments were performed using the Agilent CE System, the Agilent G3250AA LC/MSD TOF System, the Agilent 1100 Series Binary HPLC Pump, the G1603A Agilent CE-MS adapter and the G1607A Agilent CE-ESI–MS Sprayer Kit (Agilent Technologies). In-house software (MasterHands) was used for data processing, quantification and peak annotation [36].

### Numerical ecology and statistical analysis

Diversity measures and statistics of the bacterial populations were analyzed using R, PAST3, and SPSS 26.0 (IBM Corporation, Armonk, NY, USA). All data are presented as mean ± SD. Alpha-diversities of bacterial compositions, as indicated by Shannon indices, were estimated using PAST. The fecal amplicon sequencing analysis was performed using taxa detected in 50% of the subjects. Data were analyzed using the unpaired t-test/Mann–Whitney U-test for comparison between HC and IBS patients due to normal/non-normal distributions of some/other variables. Wilcoxon signed-rank test was applied for changes from IBS-n to IBS-s. The significance level threshold was set as *P* < 0.05. Graphs were plotted using the R ggplot2 package.

## Results

None of the subjects had severe caries, periodontal disease, or other dental disorders. Paired oral and fecal microbiota samples from 40 HC and 43 IBS patients (IBS-n and IBS-s) were analyzed. Within the IBS subject analysis, patients with exacerbation of IBS symptoms exhibited a significant increase in abdominal pain compared with IBS patients without symptom exacerbation (*P* < 0.01). The IBS-SI scores were significantly higher in patients with IBS than in HC (IBS, 188.7 ± 65.4 vs. HC, 38.0 ± 38.7; *t* (81) = 12.66; *P* < 0.001; Cohen’s *d* = 2.78; 95% confidence interval [CI], 127.07 to 174.47). There were no significant differences in state anxiety (IBS, 41.2 ± 10.1 vs. HC, 37.5 ± 9.2; *t* (81) = 1.74; *P* = 0.09; Cohen’s *d* = 0.38; 95% CI, − 0.53 to 7.91) or SDS scores (IBS, 37.8 ± 6.6 vs. HC, 35.2 ± 7.3; *t* (81) = 1.74; *P* = 0.09; Cohen’s *d* = 0.38; 95% CI, − 0.38 to 5.71) between IBS patients and HC, except trait anxiety (IBS, 46.8 ± 8.3 vs. HC, 41.2 ± 9.0; *t* (81) = 3.00; *P* = 0.004; Cohen’s *d* = 0.65; 95% CI,  1.85 to 9.43).

### Amplicon sequencing of fecal microbiota

**Fig. 1:**
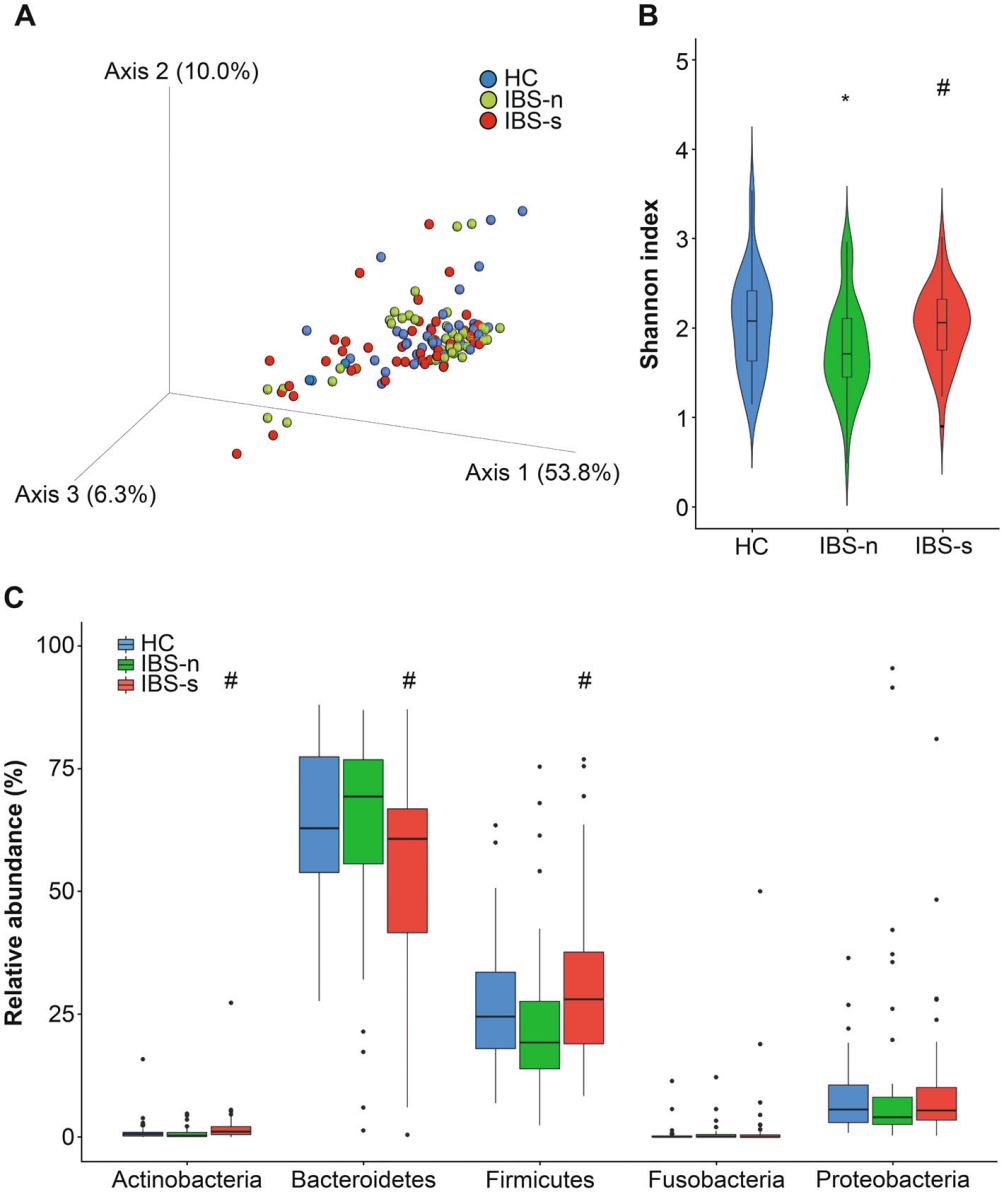
Diversity and taxonomic analyses of the fecal microbiota through 16S rRNA gene sequencing in IBS patients with and without symptom exacerbation and healthy controls. **A** Principal coordinate analysis (PCoA) of weighted UniFrac distances between healthy controls (*n* = 40) and IBS patients with and without symptom exacerbation (*n* = 43). PCoA showed no significant differences between IBS patients with and without symptom exacerbation. **B** The alpha-diversity by Shannon index. **C** Phylum relative abundances in feces. HCs, healthy controls; IBS-n, IBS without symptom exacerbation; IBS-s, IBS with symptom exacerbation. Results are expressed as means ± SD. *P < 0.05 compared with HC, ^#^*P* < 0.05 compared with IBS-n, Mann–Whitney *U*-test and Wilcoxon signed-rank test

The 16S rRNA gene sequencing analysis results from the fecal microbiota were compared using the fecal α-diversity and analyzed using Shannon indices. There were significant differences between the HC samples and IBS-n samples (*P* = 0.02) and between IBS-n and IBS-s samples from IBS-D patients (*P* < 0.01) (Fig. 1).

The taxonomic composition of the fecal microbiota demonstrated no significant differences between HC and IBS-n. At the phylum level, IBS-s exhibited significant increases in the relative abundance of Actinobacteria (*P* < 0.01) and Firmicutes (*P* = 0.01) compared to that IBS-n. Bacteroidetes (*P* = 0.01) decreased significantly in IBS-s compared with IBS-n. At the class level, analyses were performed in significantly increased phyla. Patients with IBS-s showed significant increases in the relative abundances of Bacilli (*P* = 0.04), Clostridia (*P* = 0.03), and Erysipelotrichia (*P* < 0.01) and significant decreases in Bacteroidia (*P* = 0.01) compared to abundances in IBS-n patients. According to the significant substances in the hierarchical order, at the species level, *Bifidobacterium longum* increased significantly in IBS-s compared to IBS-n (*P* < 0.01) (Table 1).

**Table 1:** Fecal phylogenetic difference between with and without symptomatic exacerbation within IBS patients

Phylum	No exacerbation %, mean (SD)	Exacerbation %, mean (SD)	*P* value	Class	No exacerbation %, mean (SD)	Exacerbation %, mean (SD)	*P* value	Order	No exacerbation %, mean (SD)	Exacerbation %, mean (SD)	*P* value
Actinobacteria	0.8 (1.1)	2.2 (4.2)	< 0.01	Actinobacteria	0.7 (1.1)	2.1 (4.2)	< 0.01	Bifidobacteriales	0.7 (1.1)	2.1 (4.2)	< 0.01
Bacteroidetes	63.1 (21.0)	54.3 (21.3)	0.01	Bacteroidia	63.1 (21.0)	54.3 (21.3)	0.01	Bacteroidales	63.1 (21.0)	54.3 (21.3)	0.01
Firmicutes	23.7 (15.7)	31.1 (17.4)	0.01	Clostridia	18.0 (11.9)	22.8 (13.9)	0.03	Clostridiales	18.0 (11.9)	22.8 (13.9)	0.03
				Bacilli	0.5 (1.1)	1.1 (2.6)	0.04	Lactobacillales	0.5 (1.1)	1.1 (2.6)	0.04
				Erysipelotrichia	0.13 (0.5)	0.5 (1.4)	< 0.01	Erysipelotrichales	0.1 (0.2)	0.5 (1.4)	< 0.01
				Negativicutes	5.0 (6.6)	6.6 (10.2)	0.05				

Family	No exacerbation %, mean (SD)	Exacerbation %, mean (SD)	*P* value	Genus	No exacerbation %, mean (SD)	Exacerbation %, mean (SD)	*P* value	Species	No exacerbation %, mean (SD)	Exacerbation %, mean (SD)	*P* value
Bifidobacteriaceae	0.7 (1.1)	2.0 (4.2)	< 0.01	*Bifidobacterium*	0.7 (1.1)	2.0 (4.2)	< 0.01	*Bifidobacterium longum*	0.1 (0.2)	0.4 (0.9)	< 0.01
Bacteroidaceae	56.3 (21.6)	49.5 (20.5)	0.04	*Bacteroides*	56.3 (21.6)	49.5 (20.5)	0.04				
Tannerellaceae	1.8 (2.4)	1.6 (2.4)	0.03	*Parabacteroides*	1.8 (2.4)	1.6 (2.4)	0.03	*Parabacteroides merdae*	1.7 (2.4)	1.5 (2.4)	.29
Marinifilaceae	0.3 (0.4)	0.2 (0.3)	0.06								
Rikenellaceae	1.1 (1.6)	0.7 (1.2)	.10								
Lachnospiraceae	10.1 (7.0)	14.7 (9.0)	< 0.01	*Agathobacter*	0.9 (1.1)	1.5 (2.3)	0.04				
Streptococcaceae	0.4 (1.0)	0.8 (1.9)	.20	*Anaerostipes*	0.7 (1.4)	0.9 (1.1)	0.06				
Peptostreptococcaceae	0.3 (0.5)	1.5 (6.2)	.93	*Blautia*	1.6 (1.4)	3.1 (2.9)	< 0.01				
Ruminococcaceae	7.3 (5.7)	6.3 (4.8)	.44	*Dorea*	0.2 (0.3)	0.6 (1.0)	< 0.01				
				*Fusicatenibacter*	0.7 (1.0)	1.0 (1.1)	0.01				
				*Lachnoclostridium*	1.2 (1.1)	1.2 (1.1)	.73				
				*Lachnospira*	0.8 (1.1)	0.4 (0.6)	0.02				
				*Roseburia*	0.4 (0.9)	0.5 (1.3)	.21				
				*Eubacterium hallii*	0.4 (0.6)	0.7 (0.9)	0.03				
				*Eubacterium ventriosum*	0.2 (0.3)	0.2 (0.4)	.40				
				*Ruminococcus gnavus*	0.5 (0.9)	1.3 (2.1)	< 0.01				
				*Ruminococcus torques*	0.5 (0.9)	1.0 (1.7)	.14				
Erysipelotrichaceae	0.1 (0.2)	0.5 (1.4)	< 0.01								

The relative abundances at the lower taxonomic levels were analyzed, when there was a significant difference in the level, Mann–Whitney *U*-test

*SD* standard deviation

### Metatranscriptome analysis of fecal samples

**Fig. 2:**
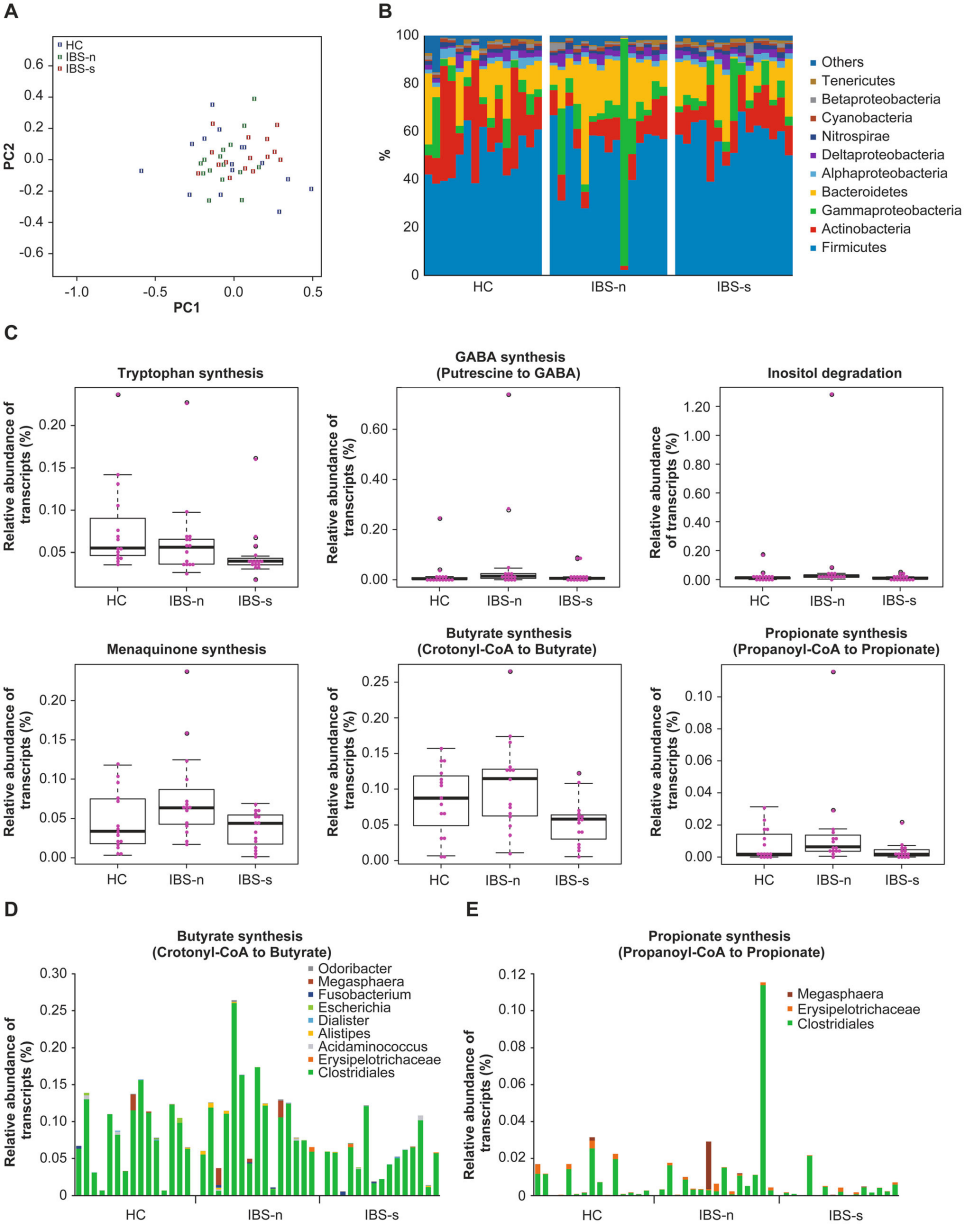
Taxonomic and functional profiles and clades in the fecal metatranscriptome. **A** A non-metric multidimensional scaling (NMDS) plot of phylum composition in metatranscriptome data. Differences in phylum compositions of 45 samples (*n* = 15 per group), which were inferred from transcript abundances of 35 universal single-copy genes, were plotted using the NMDS method in R (metaMDS function in the vegan package). The stress of the NMDS result was 0.20. The color of each dot indicates the healthy controls (HC), IBS without symptom exacerbation (IBS-n), and IBS with symptom exacerbation (IBS-s). **B, C** Box plots of significantly different MGB transcript abundances between HC, IBS-n, and IBS-s. The bold lines in the boxes indicate median values. The vertical axis indicates the relative abundance of transcripts against the total abundance of KOs-assigned transcripts. **D** Taxonomic composition of transcripts in MGB053 (butyrate synthesis). Because some taxa of Clostridiales and Erysipelotrichaceae were difficult to accurately classify at the genus level, we used a Clostridiales order and an Erysipelotrichaceae family for this graph. **E** Taxonomic composition of transcripts in MGB054 (propionate synthesis). Because some taxa of Clostridiales and Erysipelotrichaceae were difficult to accurately classify at the genus level, we used a Clostridiales order and an Erysipelotrichaceae family for this graph

A metatranscriptome analysis in 15 patients with IBS with high IBS-SI and abdominal pain scores and 15 randomly-selected HC was conducted to evaluate the active biological contribution of metabolites from the microbial communities. The total number of contigs was 679,558 sequences with a minimum of 200 bp and a maximum of 84,736 bp. In these contigs, 973,347 protein-coding genes were predicted. Phylum compositions of 45 samples, which were inferred from transcript abundances of universally single-copy genes, indicated that the phylum compositions of control samples were different from those of the IBS-n samples (Fig. 2, *P* < 0.05, PERMANOVA). In particular, the transcript abundances of the Actinobacteria phylum in the IBS-n samples were significantly lower than those of samples from HC (*P* < 0.01, Mann–Whitney *U*-test), but not significantly different from those of IBS-s (*P* = 0.18, Wilcoxon signed-rank test).

We compared the gene expressions of 56 MGB with suspected involvement in IBS pathophysiology [33]. The transcription levels of six genes involved in enzymatic glutamine to tryptophan synthesis, putrescine to GABA synthesis, inositol degradation, menaquinone synthesis, crotonyl-CoA to butyrate synthesis, and propionate synthesis were significantly different among the groups/conditions (Fig. 2, *P* < 0.05, Kruskal–Wallis test). Among these six MGBs, the transcription levels of all the enzyme genes were significantly lower in IBS-s compared with IBS-n (*P* < 0.05, Wilcoxon signed-rank test), except for the gene encoding for the enzyme in glutamine to tryptophan synthesis (MGB005). Wilcoxon signed-rank test showed these *P*-values; inositol degradation *P* = 0.01, butyrate synthesis II *P* < 0.01, tryptophan synthesis *P* = 0.39, GABA synthesis III *P* = 0.03, menaquinone synthesis *P* = 0.04, propionate synthesis II *P* = 0.03. The largest difference was observed in the transcription level (approximately two-fold changes) of crotonyl-CoA to butyrate synthesis (MGB053). MGB genes derived from Clostridiales were abundantly expressed in almost all samples (Fig. 2). Therefore, the butyrate synthesis activity of Clostridiales may have been lower in IBS-s samples compared with IBS-n samples (Fig. 2). The genera associated with the five MGBs in Clostridiales were diversified in the samples. Thus, multiple genera may have been involved in these pathways.

### Metabolome analysis of fecal samples

**Fig. 3:**
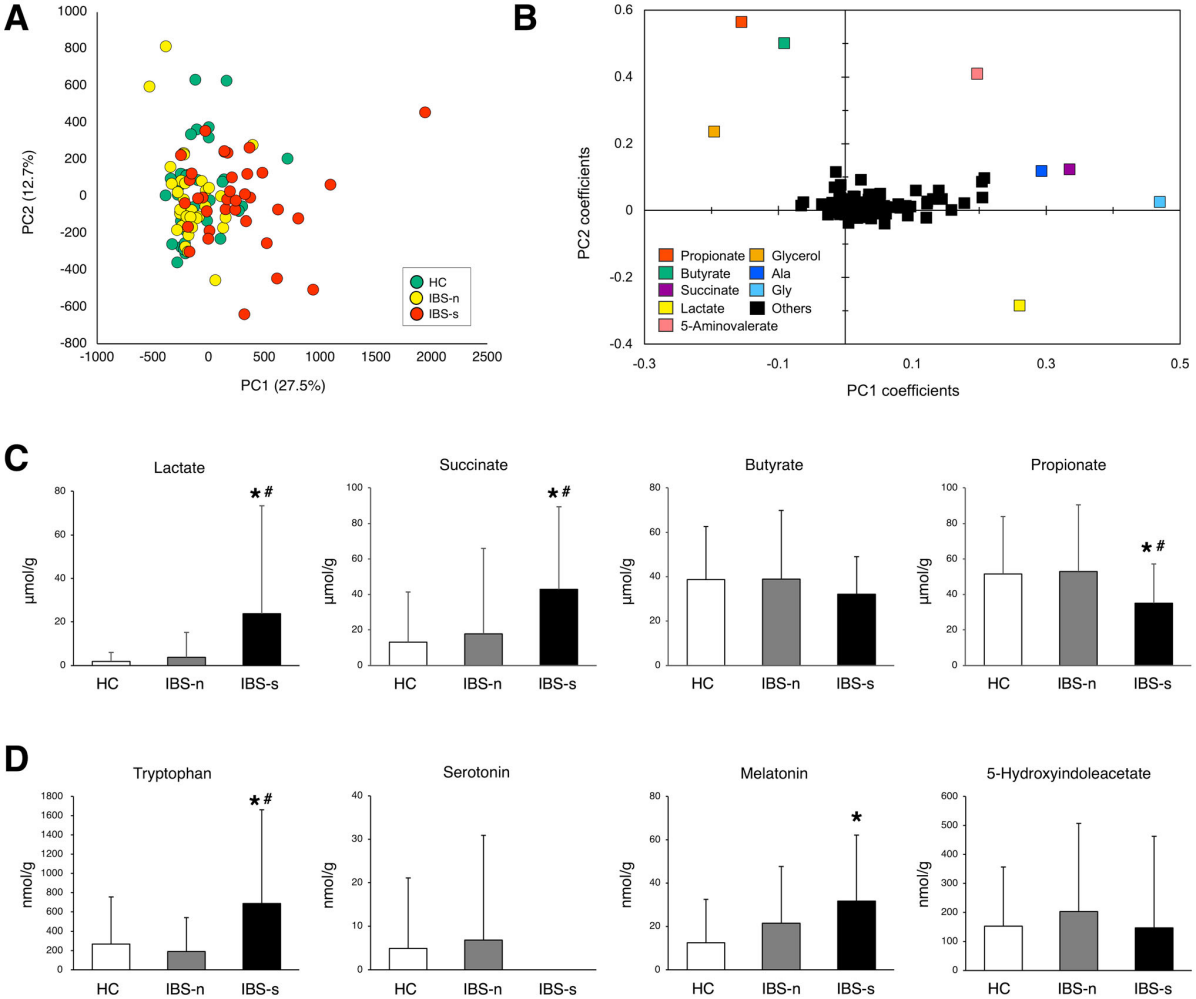
Fecal metabolome features of IBS patients with and without symptom exacerbation and healthy controls. **A** PCA showed no significant differences between healthy controls (HC, *n* = 39), IBS patients without symptom exacerbation (IBS-n, *n* = 35), and IBS patients with symptom exacerbation (IBS-s, *n* = 34). **B** PCA-derived score plots based on relative levels of identified metabolites. **C** Amounts of short-chain fatty acid (SCFA) per gram dried feces between the three sample types were compared. **D** Amounts of tryptophan, serotonin, melatonin and 5-hydroxyindoleacetate. Metabolites in the biosynthetic pathways of tryptophan-serotonin to melatonin or to the oxidative serotonin metabolite, 5-hydroxyindoleacetate, were compared. Results are expressed as means ± SD. *P < 0.05 compared with HC, ^#^*P* < 0.05 compared with IBS-n, Mann–Whitney *U*-test and Wilcoxon signed-rank test

PCA plots of fecal metabolomics data focused on subjective group projection failed to show any separation between HC, IBS-n, and IBS-s (Fig. 3). The major end-products from microbial fermentation were SCFAs; hence, a PCA based on variable projection for SCFAs was performed. Propionate, butyrate, lactate, and succinate showed discrimination (Fig. 3). Lactate (*P* < 0.01) and succinate (*P* < 0.01) levels were higher and propionate (*P* < 0.05) levels were lower in IBS-s samples compared with IBS-n samples. No significant differences in propionate, butyrate, lactate, and succinate levels were observed when HC and IBS-n subjects were compared.

We assessed the production of neurometabolite components and the degradation process. In the glutamate-glutamine-GABA cycle, glutamine (*P* < 0.01) and GABA levels (*P* = 0.02) increased in IBS-s samples compared with these levels in IBS-n samples. While glutamate was not significantly different between IBS-n and IBS-s samples, IBS-n samples showed significantly lower levels compared to HC samples (Supplementary Fig. 1). In the serotonin biosynthetic pathway, tryptophan concentration was significantly higher in IBS-s samples compared with the concentration in IBS-n samples (*P* < 0.01). Although melatonin levels were not significantly different with respect to IBS symptom exacerbation, melatonin concentration was significantly higher in IBS-s samples compared to the concentration in HC samples (*P* < 0.01). There was no significant difference in tryptophan, serotonin, melatonin and 5-hydroxyindoleacetate between HC and IBS-n samples (Fig. 3).

### Oral microbiome analysis

The taxonomic compositions of plaque microbiota exhibited more Fusobacteria (*P* = 0.03) and less Proteobacteria (*P* = 0.01) and the saliva microbiota showed more Firmicutes (*P* = 0.01) in IBS-n samples compared with those in HC samples (Supplementary Fig. 2). In the plaque microbiota, at the genus level, *Leptotrichia* of the phylum Fusobacteria was higher in IBS-n samples (*P* < 0.01) compared with *Leptotrichia* in HC samples (Supplementary Table 1). In the saliva microbiota, no differences were observed at the class levels for the phylum Firmicutes (Supplementary Table 2). The microbiota detected by 16S rRNA gene sequencing analysis that showed a significant increase in the stool did not show a significant increase in dental plaque or saliva.

### Metabolomic analysis of saliva samples

The PCA for the metabolic composition of SCFAs revealed that SCFAs, especially propionate, butyrate, and lactate, were likely to be involved in IBS symptom exacerbation (Supplementary Fig. 3). However, there were no significant differences in these SCFAs between HCs, IBS-n, and IBS-s. Moreover, neural metabolites were not significantly different between these groups.

## Discussion

Changes in fecal omics profiles were detected during IBS symptom exacerbation, especially in SCFA and neuroendocrine hormones, while there were no overlaps between fecal and oral microbiota. Bacteroidetes decreased in IBS-s compared to IBS-n fecal samples. Bacteroidetes can reverse mucosal dysfunction. Mucosal dysfunction involves altered tight junction permeability induced by inflammatory cytokines [37], and it may also induce alterations in mucus glycosylation [38]. Firmicutes contain some protease-producing bacteria [39]. Patients with IBS present with colonic mucosa microinflammation with increased proteases, interleukin-6 (IL-6), tumor necrosis factor-α (TNF-α), and decreased interleukin-10 (IL-10) levels [40]. Interestingly, loose stools in healthy subjects showed more abundant Bacteroidetes [19]. These findings, combined with our data, indicate that changes in fecal Bacteroidetes and Firmicutes composition may contribute to symptomatic exacerbation in IBS-D patients.

In the metabolome data, lactate and succinate levels were higher and propionate levels were lower in IBS-s compared with the levels in IBS-n. The main enzyme for propionate synthesis (MGB54) is propionate CoA-transferase [33]. This gene codes for the enzyme that catalyzes the conversion of acetate/lactate to propionate. The genes for propionate synthesis and crotonyl-CoA to butyrate synthesis were transcribed at lower levels in fecal samples from patients with IBS-s. Consistent with this finding, lactate levels were higher and propionate and butyrate levels were lower in the fecal metabolome of these patients. Thus, these two fecal omics data were in agreement. Both of the MGBs were contributed mainly by Clostridiales, indicating that the activities of Clostridiales might change with the onset of symptom exacerbation in IBS patients. Butyric acid-producing Clostridiales, such as *Clostridium butyricum*, suppress TGF-β and induce IL-10 in the intestine to improve IBS-D symptoms [41]. The phylogenetic analysis of 16S rRNA gene sequences includes spore-forming and dead bacteria; thus, our data suggest a link between fecal Clostridiales and butyrate production during symptomatic changes in IBS-D patients.

Metabolic SCFAs enhance host-microbiome interactions in IBS patients. We showed that fecal lactic and succinic acid concentrations were increased, whereas butyrate and propionate concentrations were decreased in IBS patients with symptom exacerbation. As lactic and succinic acids are upstream metabolites of carbohydrates, these metabolites may enhance colonic motility and accelerate fecal excretion during symptom exacerbation. In addition, the suppression of butyrate and propionate suggests that inflammation-related changes occurred throughout the mucosal areas [42].

A majority of organic acid fermentation processes are affected by pH. Acidic pH decreases lactic acid and succinic conversion to butyrate and propionate [43]. *Bifidobacterium* strains affect colonic fermentation and production of SCFA. Lactose concentrations decrease substantially at pH 6.7 compared to pH 6.2 or 5.7, with the addition of *Bifidobacterium longum* in vitro [44]. Moreover, *Bifidobacterium* can adapt to the presence of bile acids [45]. We found increased *Bifidobacterium* composition in fecal IBS-s samples, although the transcription abundance of Actinobacteria was lower. These findings are contradictory to reports showing the beneficial effects of *Bifidobacterium longum* oral administration [46] and the dependence of IBS symptom severity on fecal acetate and propionate concentration [47]. One possible explanation for this discrepancy is the protective role of butyrate in low-grade inflammation compared with other SCFAs [48]. Our fecal metabolome-metatranscriptome data suggest that interaction actively occurs between host colonic mucosa and fecal microbiomes, even when symptoms are not present. However, the fecal microbiome composition was not altered in IBS patients. Future studies are needed to clarify the effects of the colonic environment on symptom exacerbation.

Increased mucosal 5-HT availability and decreased serotonin transporter and 5-HT3 receptor antagonists can affect IBS-D symptoms [8]. Our data suggest positive feedback for serotonin synthesis. Interestingly, melatonin may improve abdominal pain when administered to patients with IBS [49]. Serotonin secretion from EC cells may be an early trigger of evoking IBS-D symptoms [50], while abdominal pain or discomfort may be influenced by the conversion rate of serotonin to melatonin in IBS patients [51]. Glutamine plays a non-neuroactive intermediate role in glutamate and GABA synthesis, which have excitatory and inhibitory roles in the brain. In addition, glutamine receptors were detected in intestinal mucosa and enteric neurons [52]. Interestingly, mucosal glutamine sensing also plays a protective role by increasing intracellular pH and mucus release. Several microbial strains, including *Lactobacillus*, *Streptococcus, Bacteroides,* and *Bifidobacterium,* can produce glutamine, glutamate, and GABA, which signal through glutamine receptors [6]. In *Bacteroides*, GABA and several other compounds are produced and excreted to lower pH conditions in the human gut [53]. In addition to its neurotransmitter function, glutamine improves gastrointestinal mucosal permeability in patients with IBS-D [54]. Thus, a highly active intermediate for the synthesis of glutamine might have potential functional regulation properties in patients with IBS.

Our data demonstrated IBS-specific differences in dental plaque microbiota but the oral and fecal microbiota in patients with IBS did not overlap. The phylum Fusobacteria, especially the species *Leptotrichia buccalis*, increased in the plaque microbiome of patients with IBS compared to HC. The major product of glucose fermentation in *Leptotrichia buccalis* is lactic acid [55]. Lactic acid may promote antitumor activities and modify immune responses in colonic mucosa [56]. In the gingiva, lactic acid promotes pathogenic microbiota in plaques resulting in immune deficiency. In contrast, no major differences in the salivary microbiomes between IBS-n/s and HC were detected. Chronic stress leading to salivary cortisol secretion [11] may alter the oral microenvironment, particularly in the anaerobic environment around the gingiva with abundant biofilms. Therefore, microbiota in plaques as a niche may account for the difference from those in saliva as fluid. The involvement of these microbiomes in signaling pathways influencing the colon are interesting aspects for future study.

The present study has several limitations. We recruited only male subjects to exclude gender effects. Female gonadal cycles might influence the microbiome and stool consistency [57]. In this study, diarrhea samples were collected only from patients with IBS and not from healthy subjects or those with functional diarrhea. In a fecal metagenomic analysis by Vandeputte et al. [19], increased Bacteroidetes were detected in watery stools in non-IBS healthy subjects. However, our amplicon sequencing data did not show such a trend. Although altered bile acid metabolism or absorption may be one of the causes of chronic diarrhea [58], we have no data on total and primary and secondary bile acid from our samples. We could only partially control the timing of sampling at exacerbation or no exacerbation at least 24 h apart in IBS patients. It is still unclear how much the faces reflected the state of the gastrointestinal tract at that time. Contiguous fecal sampling and omics analysis would help in understanding host cellular behavior and microbiome communication associated with consecutive symptom exacerbation of IBS.

In conclusion, our findings suggest that the colonic microbial environment is altered in patients with IBS-D with exacerbation of symptoms. Although oral microbiomes did not directly overlap with the IBS fecal microbiome profiles, the relative abundances in dental plaques in IBS-D with no symptom exacerbation were different from HC. The plaque components may act as a potential indicator of IBS-D.

## Supplementary Information

Below is the link to the electronic supplementary material.

Supplementary file1 (DOCX 18 KB)

Supplementary file2 (DOCX 53 KB)

Supplementary file3 (DOCX 32 KB)

Supplementary Fig. 1. Fecal amounts of glutamate, glutamine, and GABA in IBS patients with and without symptom exacerbation and healthy controls. Healthy controls (HC, n = 39), IBS patients without symptom exacerbation (IBS-n, n = 35), and IBS patients with symptom exacerbation (IBS-s, n = 34) were compared using Mann–Whitney U-test. Results are expressed as means ± SD. *P < 0.05 compared with HC, ^#^P < 0.05 compared with IBS-n, Mann–Whitney U-test and Wilcoxon signed-rank test (TIF 1327 KB)

Supplementary Fig. 2. Taxonomy and diversity of plaque and salivary microbiomes. (A) The alpha-diversity by Shannon index. (B) Phylum relative abundance. HCs, healthy controls; IBS-n, IBS without symptom exacerbation; IBS-s, IBS with symptom exacerbation. Results are expressed as means ± SD. *P < 0.05 compared with HC, Mann–Whitney U-test and Wilcoxon signed-rank test (TIF 3259 KB)

Supplementary Fig. 3. Principal component analysis of the salivary metabolome. PCA showing no significant differences between healthy controls (HC, n = 38), IBS patients without symptom exacerbation (IBS-n, n = 39), and IBS patients with symptom exacerbation (IBS-s, n = 40). (B) PCA-derived score plots based on relative levels of identified metabolites (TIF 1525 KB)

## Data Availability

Sequence data from this article have been deposited with DDBJ DRA (accession numbers DRA013075).

## Abbreviations

CRH: Corticotropin-releasing hormone
EC: Enterochromaffin cells
HC: Healthy control
IBS: Irritable bowel syndrome
KEGG: Kyoto Encyclopedia of Genes and Genomes
KO: KEGG Orthology
NMDS: Non-metric multidimensional scaling
PCA: Principal component analysis
PCoA: Principal coordinate analysis
SCFA: Short-chain fatty acid
SD: Standard deviation
SDS: Self-Rating Depression Scale
TPM: Transcripts per million
UPGMA: Unweighted pair group method with arithmetic mean
