# Supplementary material for: Omics profiles of fecal and oral microbiota change in irritable bowel syndrome patients with diarrhea and symptom exacerbation
Source: J Gastroenterol. 2022 Jul 30;57(10):748–60. doi: 10.1007/s00535-022-01888-2 (PMC9522833; doi:10.1007/s00535-022-01888-2)
Supplement: Supplementary file 1 — Supplementary file1 (DOCX 18 KB) [file 535_2022_1888_MOESM1_ESM.docx]

**Supplementary Material**

**Materials and methods**

**16S rDNA sequencing**

In the 16S rDNA amplicon sequencing, next-generation sequencing library preparations and Illumina MiSeq sequencing were conducted at GENEWIZ, Inc. (Suzhou, China). DNA samples were quantified using a Qubit 2.0 Fluorometer (Invitrogen, Carlsbad, CA, USA). Then, 30–50 ng DNA was used to generate amplicons using a MetaVxTM Library Preparation kit (GENEWIZ, Inc., South Plainfield, NJ, USA). V3 and V4 hypervariable regions of prokaryotic 16S rDNA were selected for generating amplicons and for subsequent taxonomy analysis. The V3 and V4 regions were amplified using forward primers containing the sequence 341F 5′– CCTACGGRRBGCASCAGKVRVGAAT–3′ and reverse primers containing the sequence 806R 5′–GGACTACNVGGGTWTCTAATCC–3′. First-round PCR products were used as templates for second-round amplicon enrichment PCR. At the same time, indexed adapters were added to the ends of the 16S rDNA amplicons to generate indexed libraries ready for downstream NGS sequencing on Illumina MiSeq. DNA libraries were validated by Agilent 2100 Bioanalyzer (Agilent Technologies, Palo Alto, CA, USA) and quantified by Qubit 2.0 Fluorometer. DNA libraries were multiplexed and loaded on an Illumina MiSeq instrument according to the manufacturer’s instructions (Illumina, San Diego, CA, USA). Sequencing was performed using a 2x250 paired-end configuration; image analysis and base calling were conducted using the MiSeq Control Software embedded in the MiSeq instrument.

**Data analysis of amplicon sequencing**

The QIIME1 data analysis package was used for 16S rDNA data analysis. The forward and reverse reads were joined and assigned to samples based on barcode and truncated by cutting off the barcode and primer sequence. Quality filtering on joined sequences was performed, and sequences that did not fulfill the following criteria were discarded: sequence length < 200 bp, no ambiguous bases, and mean quality score ≥ 20. The sequences were then compared with the reference database of chimeras (RDP Gold database) using UCHIME algorithm to detect chimeric sequences, followed by the chimeric sequences being removed. The effective sequences were used in the final analysis. Sequences were grouped into operational taxonomic units (OTUs) using the clustering program VSEARCH(1.9.6) against the SILVA 128 database preclustered at 97% sequence identity. The Ribosomal Database Project (RDP) classifier was used to assign a taxonomic name to all OTUs at a confidence threshold of 0.8. The RDP classifier uses the SILVA 128 database that has taxonomic names predicted to the species level.
