# Supplementary material for: Omics profiles of fecal and oral microbiota change in irritable bowel syndrome patients with diarrhea and symptom exacerbation
Source: J Gastroenterol. 2022 Jul 30;57(10):748–60. doi: 10.1007/s00535-022-01888-2 (PMC9522833; doi:10.1007/s00535-022-01888-2)
Supplement: Supplementary file 2 — Supplementary file2 (DOCX 53 KB) [file 535_2022_1888_MOESM2_ESM.docx]

Supplementary Table 1

Phylogenetic differences in Plaque samples between patients with asymptomatic HC and IBS-n
