# Supplementary figures and images for: Omics profiles of fecal and oral microbiota change in irritable bowel syndrome patients with diarrhea and symptom exacerbation
Source: J Gastroenterol. 2022 Jul 30;57(10):748–60. doi: 10.1007/s00535-022-01888-2 (PMC9522833; doi:10.1007/s00535-022-01888-2)

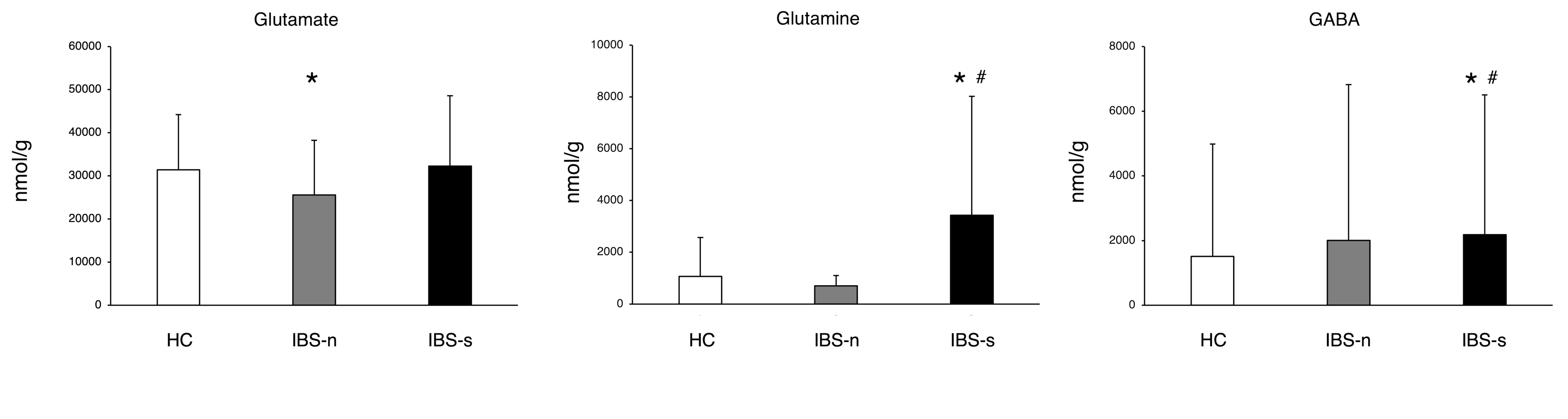

Supplement: Supplementary file 4 — Supplementary Fig. 1. Fecal amounts of glutamate, glutamine, and GABA in IBS patients with and without symptom exacerbation and healthy controls. Healthy controls (HC, n = 39), IBS patients without symptom exacerbation (IBS-n, n = 35), and IBS patients with symptom exacerbation (IBS-s, n = 34) were compared using Mann–Whitney U-test. Results are expressed as means ± SD. *P < 0.05 compared with HC, #P < 0.05 compared with IBS-n, Mann–Whitney U-test and Wilcoxon signed-rank test (TIF 1327 KB) [file 535_2022_1888_MOESM4_ESM.tif]

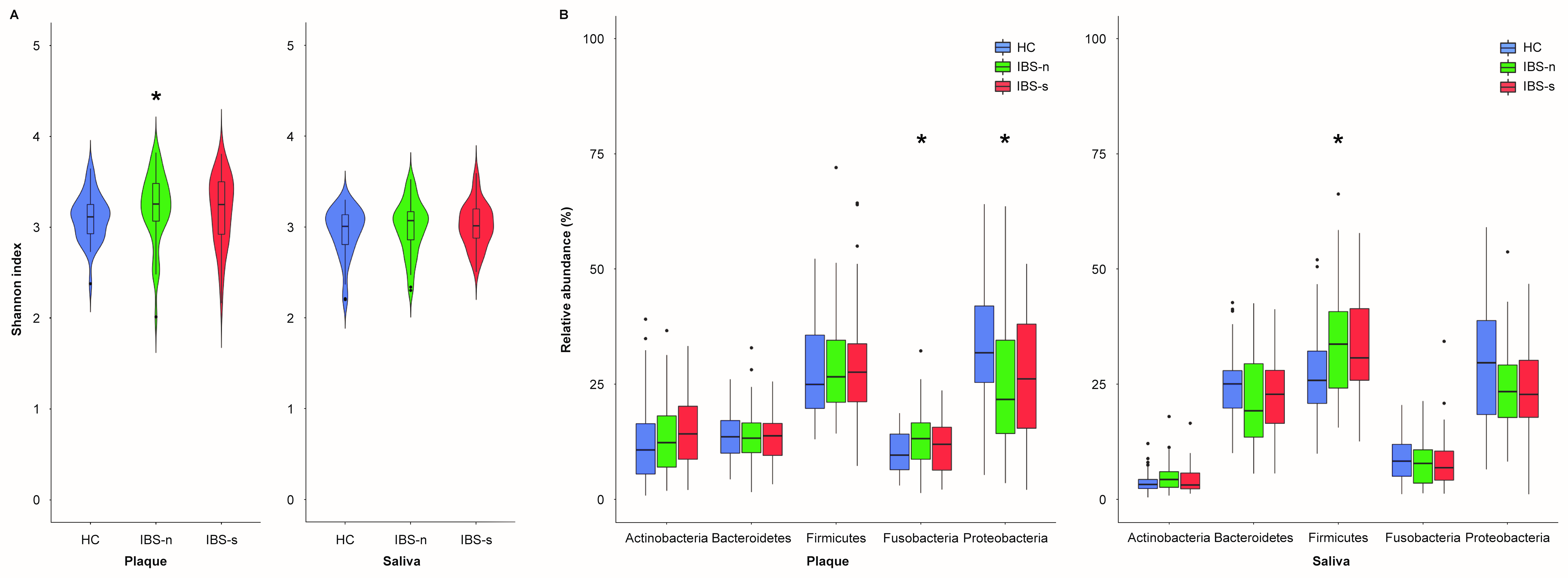

Supplement: Supplementary file 5 — Supplementary Fig. 2. Taxonomy and diversity of plaque and salivary microbiomes. (A) The alpha-diversity by Shannon index. (B) Phylum relative abundance. HCs, healthy controls; IBS-n, IBS without symptom exacerbation; IBS-s, IBS with symptom exacerbation. Results are expressed as means ± SD. *P < 0.05 compared with HC, Mann–Whitney U-test and Wilcoxon signed-rank test (TIF 3259 KB) [file 535_2022_1888_MOESM5_ESM.tif]

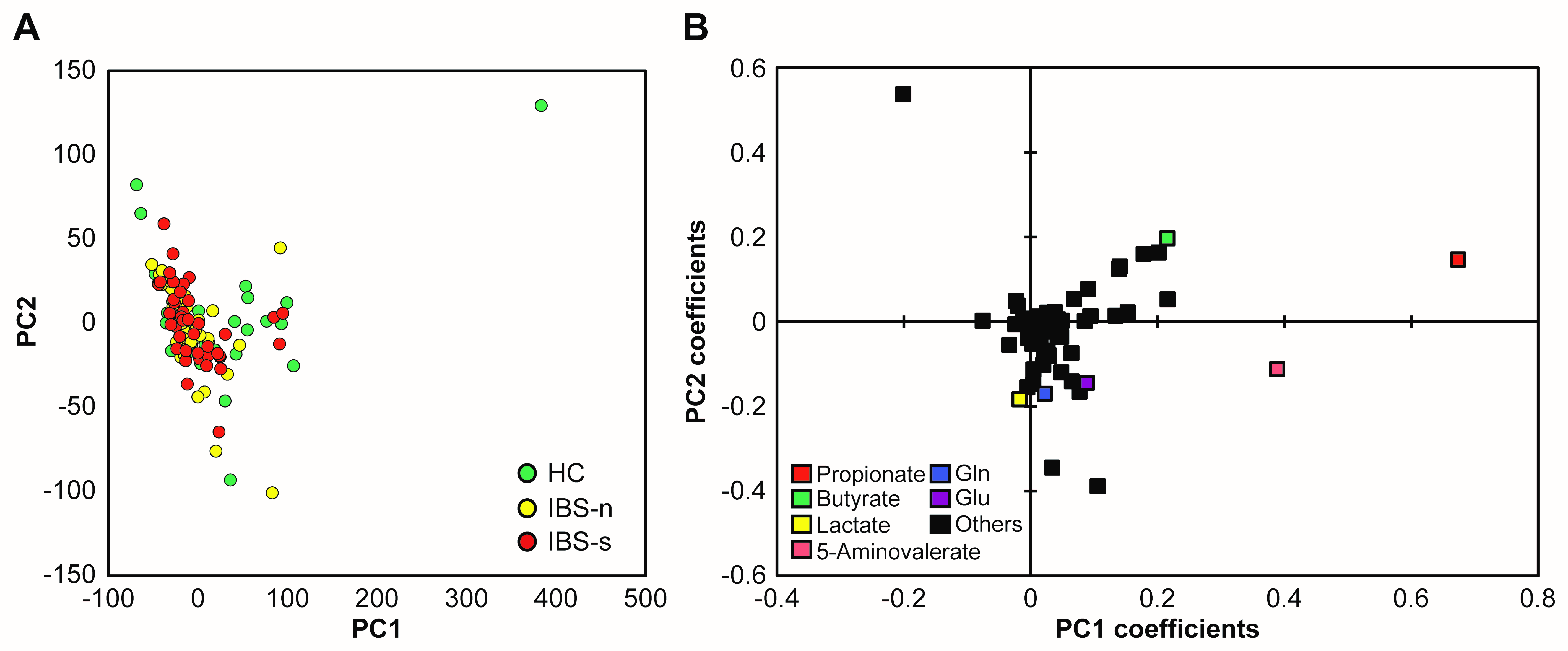

Supplement: Supplementary file 6 — Supplementary Fig. 3. Principal component analysis of the salivary metabolome. PCA showing no significant differences between healthy controls (HC, n = 38), IBS patients without symptom exacerbation (IBS-n, n = 39), and IBS patients with symptom exacerbation (IBS-s, n = 40). (B) PCA-derived score plots based on relative levels of identified metabolites (TIF 1525 KB) [file 535_2022_1888_MOESM6_ESM.tif]
